# A linkage map of the Atlantic salmon (*Salmo salar*) based on EST-derived SNP markers

**DOI:** 10.1186/1471-2164-9-223

**Published:** 2008-05-15

**Authors:** Thomas Moen, Ben Hayes, Matthew Baranski, Paul R Berg, Sissel Kjøglum, Ben F Koop, Willie S Davidson, Stig W Omholt, Sigbjørn Lien

**Affiliations:** 1 CIGENE – Centre of Integrative Genetics, Ås, Norway; 2 AKVAFORSK – Institute of Aquaculture Research, Ås, Norway; 3 Institute of Animal and Aquacultural Sciences, Norwegian University of Life Science, Ås, Norway; 4 Aqua Gen AS, Trondheim, Norway; 5 Centre for Biomedical Research, Department of Biology, University of Victoria, Victoria, British Columbia, Canada; 6 Department of Molecular Biology and Biochemistry, Simon Fraser University, Burnaby, British Columbia, Canada

## Abstract

**Background:**

The Atlantic salmon is a species of commercial and ecological significance. Like other salmonids, the species displays residual tetrasomy and a large difference in recombination rate between sexes. Linkage maps with full genome coverage, containing both type I and type II markers, are needed for progress in genomics. Furthermore, it is important to estimate levels of linkage disequilibrium (LD) in the species. In this study, we developed several hundred single nucleotide polymorphism (SNP) markers for the Atlantic salmon, and constructed male and female linkage maps containing SNP and microsatellite markers. We also investigated further the distribution of male and female recombination events across the genome, and estimated levels of LD between pairs of markers.

**Results:**

The male map had 29 linkage groups and was 390 cM long. The female map had 30 linkage groups as was 1983 cM long. In total, the maps contained 138 microsatellite markers and 304 SNPs located within genes, most of which were successfully annotated. The ratio of male to female recombination events was either close to zero or very large, indicating that there is little overlap between regions in which male and female crossovers occur. The female map is likely to have close to full genome coverage, while the majority of male linkage groups probably lack markers in telomeric regions where male recombination events occur. Levels of r^2 ^increased with decreasing inter-marker distance in a bimodal fashion; increasing slowly from ~60 cM, and more rapidly more from ~12 cM. Long-ranging LD may be consequence of recent admixture in the population, the population being a 'synthetic' breeding population with contributions from several distinct rivers. Levels of r^2 ^dropped to half its maximum value (above baseline) within 15 cM, and were higher than 0.2 above baseline for unlinked markers ('useful LD') at inter-marker distances less than 5 cM.

**Conclusion:**

The linkage map presented here is an important resource for genetic, comparative, and physical mapping of the Atlantic salmon. The female map is likely to have a map coverage that is not far from complete, whereas the male map length is likely to be significantly shorter than the true map, due to suboptimal marker coverage in the apparently small physical regions where male crossovers occur. 'Useful LD' was found at inter-marker distances less than 5 cM.

## Background

The Atlantic salmon (*Salmo salar*) is a species of worldwide significance as a prized species in recreational fishing and a major contributor to the world's aquaculture production. The genomes of the Atlantic salmon and other salmonids are purported to be derivates of an autotetraploidisation event that occurred in their common ancestor 25 to 100 million years ago (reviewed in [1]). The subsequent re-diploidisation process is not yet complete, and is illustrated in such phenomena as duplicated DNA markers (e.g. [1]); the formation of tetravalent complexes during male meioses [2]; the apparent linkage of non-linked loci due to non-random dissociation of the tetravalent complexes (pseudolinkage) [3,4]; unusual, partly tetrasomic, segregation patterns [1,5-7]; as well as chromosome arm numbers twice that of most other fin-fish species (reviewed in [8]). Furthermore, the formation of tetravalent complexes in males is believed to be the cause of another phenomenon observed in salmonids, very large differences in recombination rates between males and females that vary according to chromosomal region [6,9-13].

Two low-density maps have been published for Atlantic salmon [10,14], in addition to the SALMAP map, a higher-density, female microsatellite map made available online [15]. This latter map is developed from segregation data from two females from the river Tay in Scotland [12,16], and contains ~700 microsatellite type I and type II markers, out of which approximately 200 have been linked to BACs in the physical map [17]. Further progress in Atlantic salmon genomics relies on these existing maps being expanded with additional markers; in particular with Single Nucleotide Polymorphism (SNP) markers, since these are the most frequent polymorphisms in addition to being the markers of choice for high-throughput genotyping. Of particular value are SNP markers located within transcribed regions, to create more links between the genetic and physical maps.

Here, we provide an update of an ongoing project aiming at detecting, testing, and mapping large numbers of EST-derived SNP markers in Atlantic salmon [18,19]. We also present a SNP/microsatellite map to be used as a framework map, onto which additional SNP markers can later be added. Furthermore, we present more detailed results on sex-specific differences in recombination rates than has been provided before for this species. Finally, we report on levels of linkage disequilibrium (LD) between markers in the aquaculture strain from whence the mapping parents were sampled. Levels of LD vary between species and between populations [20-26], and have major implications for the feasibility of performing e.g. association studies and for fine-mapping Quantitative Trait Loci (QTL).

## Results

### Detection and testing of SNP markers

Hayes et al. [18] previously described the *in silico *detection of a large number of putative SNPs for Atlantic salmon, and the subsequent experimental testing of 86 of these SNPs in a diverse validation panel. In the present study, another set of 1369 SNP markers were tested in a similar validation panel (Table 1). Of the 1369 SNPs, 668 were polymorphic, reliably scored, and non-duplicated (Table 2). Of the 668 polymorphic, non-duplicated SNPs, 307 were chosen to be genotyped in the mapping families. These 307 SNPs had an average minor allele frequency of 0.27, and an average observed heterozygosity of 0.29 (results for individual SNPs in Additional File 1). Out of 307 SNPs, 244 (79%) resided in genes that could be identified and annotated (Additional File 1).

**Table 1:** Samples used for SNP validation

Country	River/population	No of samples
Canada	Conne	3
Canada	Placentia Bay	3
Iceland	Laxá	3
Iceland	Leirvogsá	3
Ireland	Moy	3
Ireland	Suir	3
Norway	Aqua Gen^a,b^	4
Norway	Byglandsfjorden^b^	4
Norway	Numedalslågen	3
Norway	SalmoBreed^a^	3
Norway/Finland	Tana-Jiesjokka	3
Russia	Neva	3
Russia	Varzuga	3
Spain	Asou	3
Spain	Pas	3

^a^Aquacultural population

^b^The Aqua Gen and Byglandsfjorden populations came in the form of 8 F1 hybrids between the two.

**Table 2:** Experimental validation of SNPs

SNP type	No.	Freq.
Normal	668	48.8%
MSV	47	3.4%
PSV	67	4.9%
All homozygous	379	27.7%
Failed	208	15.2%
Total	1369	100.0%

SNPs in the "normal" category were polymorphic, reliably scored and non-duplicated. MSV (multiple sequence variant) [49] SNPs were likely duplicated with polymorphism at one or both loci, PSV (paralogous sequence variants) SNPs were heterozygous in all animals, and thus likely duplicated without polymorphism. Failed SNPs displayed poor clustering of genotypes and/or unreliably scored genotypes.

### Linkage map

The mapping families, 10 full-sib families from a commercial breeding company, were genotyped for 307 SNPs and for 146 microsatellite markers. Out of the 307 SNP markers, 304 were polymorphic in at least one mapping parent. The SNPs belonged to 263 contigs, 222 of these having one SNP, 36 having two SNPs, and five having three SNPs.

**Figure 1:**
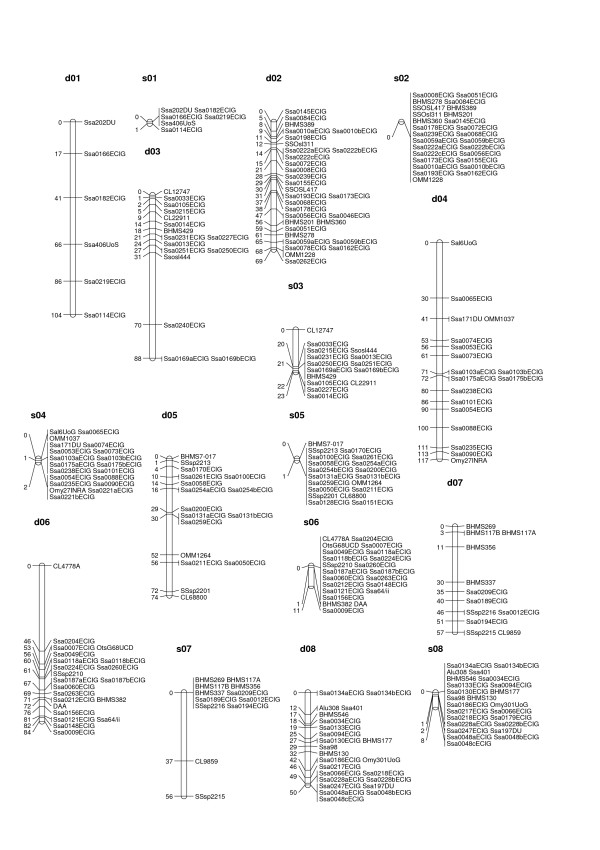
**male and female linkage maps for Atlantic salmon (linkage groups d01 to s08)**. Male and female linkage groups have prefixed s- and d-respectively. The linkage group nomenclature (numbers) is the same as in the map developed by the SALMAP project [15], except for one linkage group (sA/dA) that contains no markers present on the SALMAP map. The map units are Kosambi cM.

**Figure 2:**
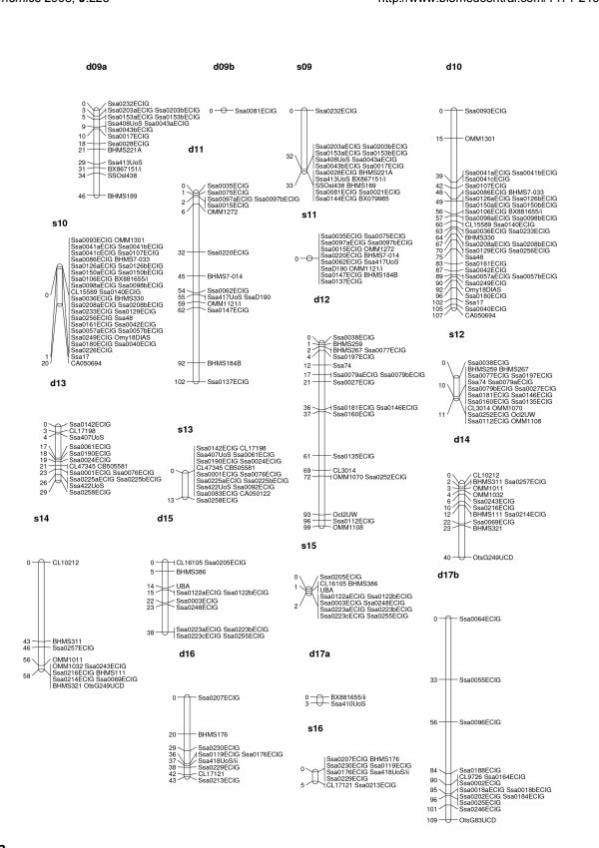
**male and female linkage maps for Atlantic salmon (linkage groups d09a to s17b)**. Male and female linkage groups have prefixed s- and d-respectively. The linkage group nomenclature (numbers) is the same as in the map developed by the SALMAP project [15], except for one linkage group (sA/dA) that contains no markers present on the SALMAP map. The map units are Kosambi cM.

**Figure 3:**
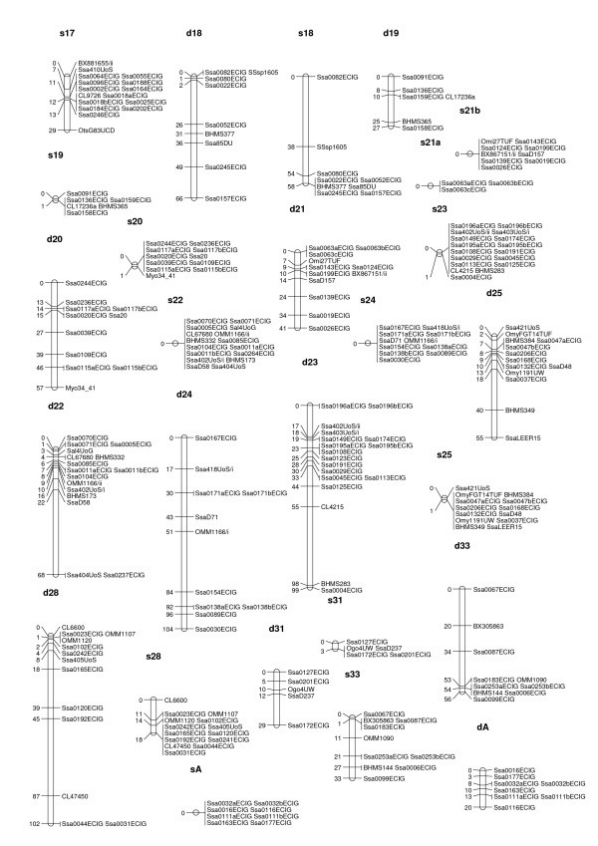
**male and female linkage maps for Atlantic salmon (linkage groups s17 to dA)**. Male and female linkage groups have prefixed s- and d-respectively. The linkage group nomenclature (numbers) is the same as in the map developed by the SALMAP project [15], except for one linkage group (sA/dA) that contains no markers present on the SALMAP map. The map units are Kosambi cM.

All but four of the informative SNPs were integrated into the map, as were all 138 informative microsatellite markers. The male and female maps consisted of 29 and 30 linkage groups, respectively (Figures 1 to 3, Table 3, Additional File 2). The male map was 390 cM long with 434 markers in total, while the female map was 1983 cM long with 425 markers in total. Lengths of linkage groups ranged from 0 cM to 59.7 cM on the male map, and from 19.8 cM to 117.1 cM on the female map (Table 3). For the most part the same linkage groups were identified for the male and the female, with the following exceptions i) two linkage groups on the male map (s9 and s17) that each corresponded to two linkage groups on the female map, and ii) one linkage group on the female map (d21) that corresponded to two linkage groups on the male map. For 57 of all possible marker pairs informative in both sexes, the orientation of markers was not the same in the two sexes (Additional File 3). However, in all 57 cases the distance between markers on the male map was relatively small (< 1.7 cM), so that these differences most likely reflect upon minor genotyping errors or missing genotypes.

**Table 3:** Properties of linkage groups

LG	♀ meioses with *n *recombination events				♀ map length (cM)	♂ map length (cM)	Markers on ♂ map	Markers on ♂ map	♂ map marker clusters
	n = 0	*n *= 1	*n *= 2	*n *≥ 3					
1	1118	687	75	0	103.8	1.21	6	6	1
2	1090	671	108	11	68.4	0	26	23	1
3	1473	390	17	0	87.6	23	15	14	2
4	861	787	231	1	117.1	2.4	16	17	1
5	1065	759	56	0	74.3	1.4	15	17	1
6	1269	560	48	3	83.5	11.3	20	20	2
7	1248	630	2	0	56.8	56.4	12	12	2
8	1416	445	18	1	49.7	8.4	20	21	2
9^♂^	NA	NA	NA	NA	NA	32.6	NA	16	2
9a^♀^	1236	577	66	1	46.1	NA	12	NA	NA
9b^♀^	NA	NA	NA	NA	NA	NA	1	NA	NA
10	1014	717	146	3	107.1	20.1	29	30	2
11	1139	601	139	1	101.9	0	14	14	1
12	767	907	206	0	99.3	10.8	18	18	2
13	1589	291	0	0	29.4	12.6	13	29	2
14	1273	581	25	1	40.2	57.8	12	12	2
15	1638	242	0	0	38.4	1.5	9	9	1
16	1661	214	5	0	43.2	4.6	9	9	2
17^♂^	NA	NA	NA	NA	NA	29.1	NA	15	3
17^♀^	1871	9	0	1	2.5	NA	2	NA	NA
17^♀^	1458	300	111	11	109.4	NA	13	NA	NA
18	1417	431	32	0	65.8	58.3	9	9	2
19	1691	189	0	0	26.8	1.2	6	6	1
20	1586	282	12	0	56.5	0.5	9	9	1
21^♀^	1625	240	15	0	41.4	NA	10	NA	NA
21a^♂^	NA	NA	NA	NA	NA	0	NA	1	1
21b^♂^	NA	NA	NA	NA	NA	0.1	NA	9	1
22	969	862	47	2	68.3	0	15	14	1
23	1242	595	40	3	98.7	1	16	15	1
24	1247	561	69	3	103.7	0	9	9	1
25	1263	595	22	0	55.2	1.3	12	12	1
28	1334	513	32	1	102	18.2	13	14	2
31	1815	65	0	0	29.2	3.2	5	5	1
33	1494	374	9	3	56.2	32.5	9	9	2
A	1832	35	13	0	19.8	0.3	6	6	1

^♀^Female-specific linkage groups

^♂^Male-specific linkage groups

We did not observe in our data set any certain instances of pseudolinkage, the apparent linkage of non-linked chromosomes sometimes observed in male salmonids [5]. One likely homeologous relationship that has not been reported before [12] was found; marker Ssa418/i was located on LG24, while Ssa418/ii was located on LG16.

### Difference in recombination patterns between sexes and between parents

**Figure 4:**
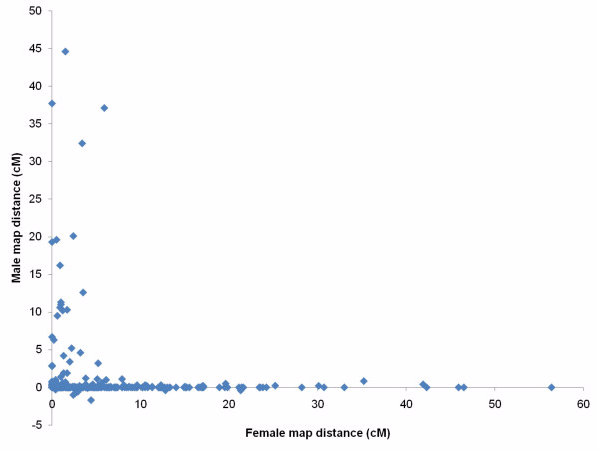
**Male and female recombination rates for pairs of adjacent markers (d09a to s17b)**. Only markers on linkage groups with a 1-to-1 relationship between the male and female homologue were considered (i.e. linkage groups 17 and 21 were excluded).

Male recombination rates were much lower than female recombination rates in large parts of the genome. In some regions, however, male recombination rates were significantly higher than those of females. Invariably, these regions were located at the end of linkage groups (Figures 1 to 3). Ratios of male to female recombination fractions for adjacent markers tended to be either close to zero or very large (Figure 4). Of the male linkage groups, 16 had all their markers grouped into one very tight cluster, 11 had one large cluster of closely linked markers located at the end of the linkage group, and one had a large central cluster bordered by markers on either side (Figures 1 to 3, Table 3).

### Linkage disequilibrium

**Figure 5:**
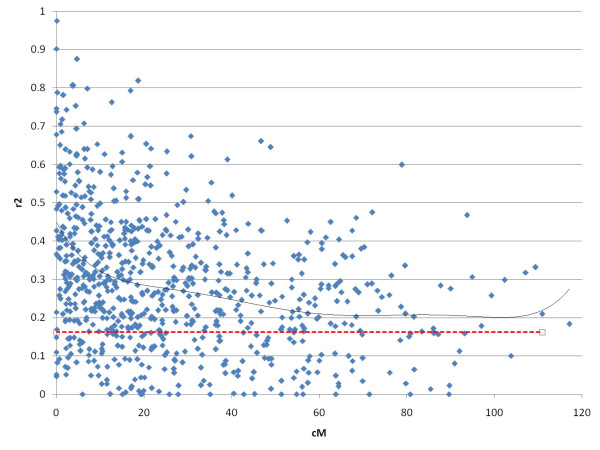
**Levels of LD between microsatellite-SNP pairs located on the same linkage group plotted versus genetic distance**. SNPs with minor allele frequencies < 0.20 and microsatellites with heterozygosity < 0.50 were excluded. Full line = 6^th ^degree polynomial best fit to the data; broken line = average level of LD between physically unlinked markers (for comparison).

Levels of LD, measured through the correlation coefficient between pairs of loci r^2^, were calculated for all microsatellite-SNP pairs with minor allele frequency (SNP) > 0.2 and heterozygosity (microsatellite) > 0.5. The average r^2 ^for marker pairs with markers located on different linkage groups was 0.16. The average r^2 ^for physically, but not genetically linked marker pairs (markers located more than 50 cM apart on the same linkage group) was 0.20. Levels of r^2 ^increased with decreasing inter-marker distance from ~60 cM, increased more rapidly more from ~12 cM (Figure 5), while r^2 ^dropped to half its maximum value (above baseline) within 15 cM.

## Discussion

### Linkage map

The male map contained 29 linkage groups, corresponding well with the most common karyotype in European Atlantic salmon, which has 2n = 58 [27]. Most likely, therefore, each male linkage group corresponds to a separate chromosome. The female map, on the other hand, contained 31 linkage groups, including two pairs of linkage groups that each corresponded to a single male homologue. Each of these two pairs is likely to correspond to a single linkage group with a large segment not covered by markers.

The lack of markers in at least two segments of the female map shows that the female map is shorter than the true female genetic map. At the same time, the low number of female-informative markers that could not be mapped, in conjunction with the relative good fit of the map length with the length of the SALMAP map (1810 cM; [15]), indicates that the female map coverage most likely is not far from complete. The male map, on the other hand, may still be considerably shorter than the true male genetic map, due to recombination events apparently being localised to a small physical region (see below).

Map distances should be expected to be slight overestimates since any genotype errors would tend to inflate genetic distances. Although practically all genotypes resulting in double crossovers were re-checked in detail and corrected if necessary, genotype errors cannot be ruled out, in particular not for markers located at the end of linkage groups (where they cannot be revealed by testing for double crossovers).

Contrary to expectation, we did not observe any instances of pseudolinkage in the data set. Pseudolinkage describes the apparent linkage in male mapping parents of markers that are not linked in female mapping parents, with an excess of non-parental genotypes in offspring, and is believed to be caused by the non-random breaking up of tetravalent complexes formed during male meiosis [5]. Pseudolinkage has earlier been found in several salmonid mapping studies [3,4,6,9,13]. In a recent study on an F1 cross between Atlantic salmon of Canadian and European origin, utilising a subset of the SNP markers described in the present study, pseudolinkage was observed between 5 pairs of linkage groups (Boulding et al., submitted). The difference in occurrence of pseudolinkage between the latter study and the present study is in line with a hypothesis stating that pseudolinkage occurs more frequently in inter-strain hybrids than in pure strain fish [5,9,28]. It should be noted, however, that our findings with respect to pseudolinkage are indicative rather than conclusive since grandparental genotypes were not available, meaning that pseudolinkage could, if present, only be detected as weak linkage in males between markers unlinked in females (and not from excess of non-parental genotypes). Also, since linkage phases were deduced from linkage analysis and not from grandparental genotyping, linkage between markers within male linkage groups could theoretically be due to pseudolinkage rather than to classical linkage. However, all markers on the male map were either i) very closely linked to other markers within the same linkage group, and hence very unlikely to be linked through pseudolinkage, or ii) appearing on the homologous linkage group in the female map. From this we conclude that pseudolinkage would be unlikely to be the cause of any observed linkages on this map.

### Difference in recombination patterns between sexes

In salmonids, male recombination rates are much reduced compared to female recombination rates [9-11,14]. In rainbow trout the ratio between male and female recombination has been found to vary considerably across linkage groups, with male recombination rates being severely depressed relative to female recombination rates in areas proximal to centromeres, but elevated in regions distal to centromeres [9]. This finding has been investigated further in rainbow trout [12], and the same phenomenon has been shown in Arctic char [13] and Atlantic salmon [10,29], though not in detail for the latter species. The results presented here support these earlier findings, but indicate that the site-specific differences in recombination rate between sexes are even more pronounced in Atlantic salmon than in rainbow trout (compare Figure 4 with Figure 2 from [9]). Since female recombination rates are much higher than male recombination rates for a large majority of adjacent marker pairs (Figures 1 to 3, Figure 4), it seems reasonable to assume that the regions where male recombination events occur are substantially smaller in physical terms than the corresponding female regions. This, again, means that, while it is likely that the female map presented here is close to full coverage, the true male map may be substantially longer than the presented male map. Higher marker densities will be needed to identify markers located in the regions where male recombination occurs, i.e. telomeric regions.

The most common karyotype of European Atlantic salmon consists of 16 metacentric and 42 acrocentric chromosomes [27]. If one assumes that male recombination occurs on all chromosomes, but only in telomeric regions, the complete male map should contain 8 linkage groups where a large central cluster of markers is surrounded by markers on both sides, and 21 groups with a large cluster at one end. In the map presented here, only linkage group 17 has a central cluster surrounded by markers on either side, indicating that the corresponding chromosome is one of the metacentric chromosomes. All other linkage groups have either only one cluster or one cluster with markers on one side, and thus may have one or two telomeric regions not covered by informative markers. Further evidence for the identification of linkage groups (i.e. chromosomes) as meta- or acrocentric could be extracted from the lengths of linkage groups (female map), and from the number of meioses with more than one recombination event (Table 3); if one assumes complete interference [30-33] then acrocentric chromosomes should have genetic lengths of ~50 cM and a small incidence of meioses with more than one crossover, while metacentric chromosomes should have genetic lengths of ~100 cM and a higher incidence of meiosis with two or more crossovers [12]. The linkage group size and number of meioses with > 2 recombinants for linkage group 17 supports this hypothesis.

The site-specific distribution of male and female recombination events must be taken into account when QTL experiments are designed. In Atlantic salmon, it has become quite common to first perform a coarse genome scan using male segregation only and one or a few markers per linkage group, on the assumption that there is practically no recombination in males ([34]; Boulding et al., submitted). While this strategy will work for most parts of the genome, QTLs located in telomeric regions (which are also gene-rich regions) will probably be missed unless markers located in these regions are added. Identification of more markers in telomeric region would therefore be highly useful.

In this study, we exploited the lack of male recombination in large parts of the genome to draw more information out of the data. More specifically, for markers located in such regions, when both parents were heterozygous and identical within a given family, heterozygous (and thus, *a priori *uninformative) offspring were assumed to have inherited the chromosome segment without recombinations from their respective fathers. In this way, alleles inherited from sire and dam could be deduced, and the markers could be re-coded as fully informative. It should be pointed out that this strategy was only used if no male recombinations had been observed in *a priori *informative offspring, meaning that no recombination had been observed in a minimum of 92 (but usually many more) meioses. The strategy was therefore conservative.

### Linkage disequilibrium

The individuals used for the LD study were a subset of the mapping parents; more specifically, 16 mapping parents that belonged to the same breeding population, the remaining four animals belonging to another population. Phase-known data were used, since haplotypes could be deduced with very high certainty due to large family sizes.

Only microsatellite-SNP pairs were used for calculation of LD, thus mimicking the mapping of a QTL (usually assumed to be bi-allelic) using microsatellites. LD was also computed between SNP-SNP pairs (results not shown), and found to be much lower than for microsatellite-SNP LD. Levels of microsatellite-microsatellite LD were comparable to those of microsatellite-SNP LD, though slightly higher (results not shown). Lower LD values for SNP-SNP pairs when compared to microsatellite-SNP or microsatellite-microsatellite pairs is likely to be, at least in part, a consequence of differences in heterozygosity between marker types [35,36]. However, it could also be due in part to higher mutation rates of microsatellites relative to SNPs, which would impact upon levels of LD (reviewed in [37]). To avoid marker pairs with low information content, SNPs with minor allele frequencies below 0.2 and microsatellites with heterozygosities below 0.5 were culled from LD analysis.

The average r^2 ^between markers located on different linkage group was higher than expected (r^2 ^= 0.16). This could be caused by limited effective population size and/or by relatedness between individuals in the sample (there were three pairs of full-sibs in the sample). However, removing closely related animals did not decrease, but rather increased r^2 ^between unlinked markers, indicating that the small sample size may have been the main reason for high LD between unlinked markers. Levels of r^2 ^is biased upwards when sample sizes are small, although much less so than the LD statistic D' [38]. At the same time, LD may have been slightly biased downward by the original detection of SNPs in EST sequences coming from a limited number of animals of both Canadian and Norwegian origin. Detection of SNPs in a small number of diverse animals could lead to an overrepresentation of old mutations among the set of SNPs, and thus to underestimation of LD between markers. It is difficult to assess the joint effect of these two factors. However, we have assumed that bias in levels of LD would not greatly affect conclusions regarding changes in LD with changing inter-marker distance.

LD between linked markers seemed to increase with decreasing inter-marker distance in a bi-modal manner. LD first increases slowly from ~60 cM, then more rapidly from ~12 cM. This finding may reflect upon the fact that the population from whence the haplotypes were derived is a 'synthetic' population, formed seven generation ago from individuals from different Norwegian rivers [39]. Due to the limited number of meioses since the formation of the base population (in this context less than four because only female crossovers would occur for most chromosomal regions), LD within linkage groups caused by population stratification would be expected to persist for long distances. Over shorter distances, LD would be caused both by population subdivision and by LD inherent in the wild population from whence the breeding population was formed. Since LD observed in the population is likely to be partly caused by population admixture, the results described here may not be relevant for wild Atlantic salmon populations. However, many breeding populations, and thus populations used for scanning of QTL/genes affecting commercial traits, are admixed in the same sense, and may thus display similar patterns of LD.

Levels of LD were calculated in order to estimate the marker densities needed to perform LD-based mapping in Atlantic salmon. The measure r^2 ^is equal to the amount of information provided by one locus about the other, meaning that for a gene-trait association to be detected, sample size must be increased by 1/r^2 ^if a marker in LD with the trait-affecting gene is used rather than the gene itself. Levels of r^2 ^from 0.1 to 0.3 have been proposed as minimum values of 'useful ld' [40,41], in which case sample sizes would have to be increased by a factor of three to 10 in order to achieve maximum power. If one accepts r^2 ^> 0.2 as the threshold, 'useful LD' could in this study be found at inter-marker distances below ~5 cM, indicating that ~400 fully informative, evenly spaced markers would be sufficient to at least start capturing inherent LD information. Thus, currently available maps ([15]; present study) could in principle provide a template for LD mapping to some extent. To fully exploit LD in the Atlantic salmon genome, however, more dense maps would probably be needed.

## Conclusion

In this study, we constructed male- and female genetic linkage maps of Atlantic salmon, elucidated further the distributions of recombination events in males and females, and provided initial data on levels of linkage disequilibrium. The female map presented here is likely to represent the true genetic map well, whereas the male map is probably incomplete due to male recombination being localised to narrow telomeric regions. There appear to be little overlap between regions in which male and female recombination events occur. Levels of LD (r^2^) were more than 0.2 above the baseline for unlinked markers at inter-marker distances less than 5 cM. At inter-marker distances larger than 15 cM, r^2 ^decreased slowly, possibly reflecting LD due to population admixture that have had limited time to be broken down by recombination. The map presented here will serve as a framework map onto which a larger number of SNP markers, currently being identified from alignment of EST sequences and from DNA re-sequencing [42], will be added.

## Methods

### SNP discovery

Putative SNP were discovered *in silico*, as described in [18]. In brief, 100,866 EST chromatograms were base-called and aligned using the software programs Phred [43] and Phrap (P. Green, unpublished), whereupon putative SNPs were identified using PolyBayes [44] and checked by manual inspection. The ESTs were derived from several individuals of the Canadian McConnell strain and from several individuals from the Aqua Gen strains [18,45,46].

### Validation and characterisation of SNPs

From among the 2507 putative SNP discovered *in silico*, 1369 were chosen to be experimentally validated based on read quality and number of reads with rare allele. These SNPs were genotyped in a panel of 47 Atlantic salmon from across the species range (Table 1). SNP genotyping was done using the MassARRAY system from Sequenom (San Diego, USA). PCR-primers and extension-primers were designed using the software SpectroDESIGNER v3.0 (Sequenom). Multiplexes and primer sequences are available on request sigbjorn.lien@umb.no. Multiplexing levels were between 20 and 29. All SNP genotyping was performed according to the iPLEX protocol from Sequenom (available at [47]). For allele separations the Sequenom MassARRAY™ Analyzer (Autoflex mass spectrometer) was used. Genotypes were assigned in real time [48] by using the MassARRAY SpectroTYPER RT v3.4 software (Sequenom) based on the mass peaks present. All results were manually inspected, using the MassARRAY TyperAnalyzer v3.3 software (Sequenom). Classification of SNPs was based on this manual inspection. The categories were: 1) *normal *= polymorphic, reliably scored and non-duplicated; 2) *MSV *multiple sequence variant) [49] = SNPs were likely duplicated with polymorphism at one or both loci (characterised by heterozygote excess, more than one cluster of heterozygotes, and presence of homozygotes); 3) *PSV *(paralogous sequence variant) = duplicated SNP without homozygotes; 4) *all homozygous *= all animals were homozygous; and 5) *failed assay *= poor clustering of genotypes and/or unreliably scored genotypes. Sequences of SNPs and contigs can be found in Additional File 1.

### Annotation of SNPs

Sequences containing SNPs were clustered into contigs using a two stage Phrap assembly process. The first stage assembly of 434,384 Atlantic salmon EST sequences (parameters 100 minmatch and 0.99 repeat_stringency) resulted in 119,912 contigs which were then reassembled (2nd stage; 300 minmatch and 0.96 repeat_stringency) into 81,398 contigs [50]. Complete contigs containing SNPs compared (BLASTX) to CDD and Swissprot databases and annotated with the top BLASTX hit if the database match had an e-value of < 10^-10^. Matches to hypothetical proteins and genomic sequences were filtered out.

### Genotyping of mapping population

The mapping families were provided by the breeding company Aqua Gen AS, and were used also for QTL mapping for resistance against the viral disease Infectious Pancreatic Necrosis (IPN) (Moen et al., in prep.). Hence, the offspring had been challenge tested for resistance to IPN. The 20 mapping parents came from two different year populations of Aqua Gen salmon; 16 from year class 2001, and 4 from year class 2000. Both populations were formed in the yearly 1970's from salmon from different Norwegian rivers [39], and have been maintained as more or less closed populations since (with increase in inbreeding per generation < 0.5%; S. Kjøglum, Aqua Gen, pers. com.). In total, 192 offspring from each of 10 full-sib families, and their parents, were genotyped. The parents of the mapping population can, in the context of the present study, be regarded as random animals sampled from the broodstock population. DNA extraction was carried out using the DNAeasy 96 kit from QIAGEN (Venlo, the Nethelands). Within each family, microsatellites were genotyped for the 96 least and the 96 most resistant animals. SNPs were genotyped on the mapping families for two reasons; 1) to position the SNPs on the linkage map, and 2) to provide more markers for eventual fine-mapping of QTL-regions. In order to achieve these two goals in an affordable manner, we chose to genotype all SNPs for IPN-resistant animals only. The animals were genotyped for 307 SNP markers and for 148 microsatellites. SNP genotyping was carried out as described above.

Most of the microsatellite markers used in this study were chosen from the SALMAP microsatellite map of Atlantic salmon (as it was in 2006) [15], and were collectively chosen to ensure good genome coverage. The microsatellite markers were distributed across 32 PCR multiplexes that were subsequently combined into 16 multiplexes for capillary electrophoresis. Primer sequences and multiplex information is available on request. Polymerase chain reactions (PCR) were performed in volumes of 5 μl, using 0.25 units of AmpliTaq Gold (Applied Biosystems), 250 μM dNTP mix, 1.5 mM MgCl_2_, 0.25 –1 pmol of each primer (depending on amplification efficiency of each marker in multiplex), 0.25 μl DMSO, and 5 ng DNA template. PCR cycling conditions were 95°C for 10 minutes, 35 cycles of 94°C for 30 seconds, 54°C for 1 minute, and 72°C for 1 minute, followed by a final extension of 60°C for 45 minutes. The lengths of the fluorescent PCR products were determined relative to the LIZ500 size standard (Applied Biosystems) on a 3730 DNA Analyzer (Applied Biosystems), using GeneMapper 4.0 (Applied Biosystems) software for allele calls.

### Construction of linkage map

Since recombination rates in salmonids have been shown to differ dramatically between sexes, separate male and female maps were constructed. Marker grouping and initial marker ordering was done in Joinmap 3.0 [51]. A Joinmap 3.0 input file was made for each mapping parent (in *double haploid *format), containing information on alleles inherited from that parent only. Marker grouping was done at a minimum LOD score of 4.0. Following marker grouping, homologous linkage groups from each sire and each dam were integrated into single sex-specific maps. The marker orders determined by Joinmap 3.0 were tested and corrected using the *flips *function of CRIMAP, with a moving window of 7 markers (*flips7*). Using the final marker orders as calculated by CRIMAP, the data was examined for unlikely double recombinants, for inconsistencies in marker order between parents, using a custom Visual Basic for Applications (VBA) for Excel program. Segregation distortion was tested for using the same program, by incorporating a Pearson's goodness-of-fit test for 1:1 segregation of alleles from individual parents to offspring. Double recombinants occurring over small distances were checked for genotyping error. Markers displaying segregation distortion (P < 0.01) were also inspected. After marker orders and potential genotype errors had been verified, the final maps were constructed using the *fixed *function of CRIMAP. The Kosambi mapping function was used. Map drawings were made using Joinmap 3.0 [51].

Since SNPs are bi-allelic, there were frequent occurrences of both parents of a family being heterozygous for the same two alleles of a SNP. In such cases, all heterozygous offspring were initially uninformative for mapping. We exploited the complete lack of male recombinants in most parts of the genome to deduce the inheritance of alleles in situations where i) both parents were heterozygous for the same two alleles of a SNP (marker A), and ii) the SNP was linked without any observed male recombinants to another marker (marker B); the latter marker being fully informative in the male. The steps of the deduction process were 1) determine the linkage phase between A and B in the male parent, 2) use the linkage phase to deduce which allele was inherited from the father at A, to offspring heterozygous at that marker, and 3) assign the other allele at A (of heterozygous offspring) to the female parent. This strategy was applied during the process of checking the data, automated through a VBA program.

Before map construction, SNP markers located within the same contig were combined to produce one marker point (i.e. a haplotype).

Linkage groups were numbered according to the SALMAP map, using shared microsatellites to infer homologies. Whenever two or more markers were shared between a linkage group and its SALMAP homologue, the linkage group was also oriented in the same way as the SALMAP linkage group. One linkage group did not share any markers with any linkage group on the SALMAP map, and was termed "A". In the cases where two unlinked (in this data set) female segments corresponded to a single male homologue X, the segments were designated Xa and Xb to indicate that they were likely to be part of the same linkage group (and *vice versa *for unlinked male segments corresponding to a single female homologue).

The *chrompic *function of CRIMAP was used to count number of recombination events per meiosis.

### Linkage disequilibrium

The animals used for calculation of LD were a subset of the mapping parents, more specifically the 16 parents that came from the breeding population of major representation. Thus, the data set consisted of 32 haplotypes. Only microsatellite-SNP pairs were used, to mimic the mapping of a QTL using microsatellite markers. SNPs with minor allele frequencies < 0.2 and microsatellites with heterozygosities < 0.5 were culled. Composite SNP markers (haplotypes of SNPs within the same contigs) were grouped with microsatellites. The haplotypes of the mapping parents were deduced at every linkage group using a custom-made VBA program. Briefly, the program performed these steps (for every linkage group within every mapping parent): 1) Start at the first informative marker from one of the linkage group; 2) find the linkage phase between that marker and the next informative marker, minimising the number of recombination events in the offspring; 3) proceed in this manner to find the linkage phase between all informative marker, and thus to build the two haplotypes; and 4) for monomorphic markers, insert the same allele in both haplotypes. Measures of LD were calculated using the function *haploxt *of the program GOLD [52]. The LD measure was the correlation coefficient, r^2^, calculated as the square of Cramer's V [53]. The sampling effect was corrected for by subtracting 1/(number of haplotypes) from r^2 ^[54]. Sved's equation [55] was fitted to the data using the *nlinfit *function of MATLAB, but provided a poor fit (as expected, since LD in the population would be expected to be caused by other factors in addition to drift). Instead, a 6^th ^degree polynomial was fitted to the data.

## Authors' contributions

TM took part in microsatellite genotyping and SNP validation, did data analyses except for SNP detection and annotation, and wrote a draft of the manuscript with contributions from BH, MB, PB, BK, WSD, BFK, and SL. BH did *in silico *SNP detection. MB coordinated microsatellite genotyping and took part in SNP genotyping. PB coordinated SNP genotyping. SK organised and carried out tissue sampling. BFK took part in annotation and provided the bulk of EST sequences. WSD took part in annotation. SWO provided laboratory facilities and took part in planning the study. SL provided laboratory facilities, did manual inspection of SNP validation data, and took part in SNP validation and data collection. All authors read and approved of the manuscript.

## Supplementary Material

Additional file 1 Properties of SNP markers used in the study. Excel workbook containing IDs of SNPs, contigs sequences w/SNP sequence and position, allele frequencies, heterozygosities, and BLASTX hits Click here for file

Additional file 2 Atlantic salmon linkage map. Linkage map in Excel format. Click here for file

Additional file 3 Marker pairs with male-female order discrepancies. Marker pairs for which marker order were inverted in one map relative to the other. Click here for file
